# Comorbid Affective and Substance Use Disorders of Medicaid/Medicare Beneficiaries at an Opioid Treatment Program Serving Small Urban and Rural Communities

**DOI:** 10.3389/fpsyt.2022.881821

**Published:** 2022-05-02

**Authors:** Jamey J. Lister, Guijin Lee, Jennifer D. Ellis, Emily Pasman, Elizabeth Agius, Stella M. Resko

**Affiliations:** ^1^Rutgers University School of Social Work, New Brunswick, NJ, United States; ^2^Wayne State University School of Social Work, Detroit, MI, United States; ^3^Johns Hopkins School of Medicine, Department of Psychiatry and Behavioral Sciences, Baltimore, MD, United States; ^4^Wayne State University Merrill Palmer Skillman Institute, Detroit, MI, United States

**Keywords:** comorbid, opioid use disorder, affective disorder, substance use disorder, methadone, Medicare and Medicaid, rural, urban

## Abstract

**Objectives:**

Identify rates and correlates of comorbid affective and substance use disorders among an understudied population, Medicaid/Medicare beneficiaries receiving care at an opioid treatment program serving patients from small urban and rural areas. Examine whether past-year non-medical opioid use status differentiates comorbidity status.

**Methods:**

A cross-sectional, venue-based design was used to recruit a convenience sample of patients treated with methadone for opioid use disorder. Measures were assessed across three domains: (1) demographic characteristics, (2) opioid use characteristics, and (3) comorbid disorders. Brief validated screeners categorized probable comorbid disorders. Bivariate analyses examined correlates of comorbid disorders and determined variable selection for multivariable analyses.

**Results:**

In this sample (*N* = 210; mean age = 38.5 years; female = 62.2%; Non-Hispanic White race/ethnicity = 86.1%), comorbid disorders were common. Rates were as follows: current anxiety (48.1%), depression (41.1%), and PTSD (33.7%), and past-year stimulant (27.6%), marijuana (19.0%), alcohol (14.9%), and sedative (7.6%). In bivariate analyses, past-year non-medical opioid use and a greater accumulation of opioid use consequences were associated with most disorders. When including demographic and opioid use characteristics in multivariable analyses, past-year non-medical opioid use was associated with anxiety, PTSD, stimulant use disorder, and sedative use disorder.

**Conclusions:**

Few studies have investigated comorbid disorders among this understudied population. This analysis highlights a high burden, especially for affective disorders. Our findings demonstrate that routine, ongoing assessment of non-medical opioid use may be a promising and feasible strategy to detect patients needing integrated care. Future research should investigate whether changes to assessment protocols at opioid treatment programs in small urban and rural settings facilitate care coordination.

## Introduction

People receiving methadone treatment for opioid use disorder (OUD) often have multiple morbidities. Studies estimate that more than 80% of patients in methadone treatment have at least one comorbid affective or substance use disorder (1, 2). Among people with OUD, past-year comorbidity rates range from 13 to 26% for alcohol, cannabis, cocaine, and sedative use disorder, and 64% have at least one mental health disorder (3). Comorbid disorders, especially other substance use disorders, are associated with worse treatment outcomes (1, 4). Comorbid depressive, trauma-related, and anxiety disorders, while less consistent risk factors for worse treatment outcomes (1, 5, 6), necessitate assessment and integrated approaches. These are especially urgent considering people with comorbid substance use and affective disorders are at high risk for fatal overdose people compared to those without comorbid disorders (7). While facilities providing methadone in the United States, formally called opioid treatment programs (OTPs), are required to provide substance use counseling or behavioral therapies in conjunction with medication treatment (8), there is no specific requirement for those counseling services to address co-occurring affective disorders (9).

Assessment and monitoring of methadone treatment processes and outcomes are required by federal guidelines for OTPs; and these guidelines recommend assessment of comorbid disorders (10). The Substance Abuse and Mental Health Services Administration (SAMHSA) advocates for the use of validated screening and assessment tools in their Treatment Improvement Protocol series on substance use and co-occurring disorders (11). However, because the use of validated tools is not a formal requirement for OTP intake, non-validated instruments developed by local and state treatment authorities are commonly used (12). Furthermore, some patients may not report psychiatric distress early in treatment (i.e., during intake assessment), but may experience symptoms that begin after induction or stabilization to methadone. Therefore, quickly screening patients at regular intervals throughout treatment may identify the need for integrated care approaches to address comorbid affective and substance use disorders. Furthermore, identifying innovative yet feasible strategies, such as low-burden screening, represents an approach that may improve care and be better adopted than resource-intensive strategies not well suited for the complexity of the existing addiction treatment system.

Screening for comorbid affective and substance use disorders in small urban and rural communities may be particularly important. People with comorbid disorders living in these settings often experience considerable challenges when trying to access care that is located outside of their OTP, due primarily to travel and transportation barriers, whether personal or public (13, 14). Research has demonstrated that OTP patients referred to care offsite are at risk for poor treatment attendance and retention (15, 16). Thus, the use of validated screening measures, which assess comorbid disorder symptoms and non-medical opioid use patterns during treatment, have the potential to improve co-located care coordination at OTPs serving small urban and rural patient populations, and address a largely unmet comorbid disorder burden.

To date, investigations gathering primary data on comorbid disorders among patients in methadone treatment are restricted mainly to samples from large urban areas, due to a scarcity of OTPs in small urban and rural areas (17, 18) and relatively few patients traveling to large urban areas from smaller surrounding communities (13). Furthermore, primary data studies of patients being treated with methadone rarely constrain the sample to target the needs of publicly-insured populations, despite disproportionate odds for methadone services to be paid by public funds (19), and higher odds of opioid overdose among Medicaid/Medicare beneficiaries (20).

This study represents the first primary data collection addressing comorbid affective and substance use disorders among a sample of Medicaid/Medicare beneficiary patients from small urban and rural communities receiving methadone treatment. Our aims were as follows. We sought to identify rates of probable comorbid affective and substance use disorders using validated screening tools; examine demographic and opioid use characteristic correlates of comorbid disorders; and investigate whether past-year non-medical opioid use status was a significant differentiator of comorbid disorder status in multivariable analyses when including other key characteristics. We hypothesized that a high rate of comorbid disorders would be observed, with stronger correlations for opioid use characteristic variables and comorbid disorders than for demographic characteristics and comorbid disorders. Last, we hypothesized that past-year non-medical opioid use status would be the most consistent correlate of comorbid disorders in multivariable analyses.

## Materials and Methods

### Setting and Procedure

A venue-based recruitment strategy was used to recruit patients receiving methadone for OUD at an OTP situated in a medically underserved area of a small urban county (Rural Urban Continuum Code/RUCC = 3) (21). The clinic's catchment area extends to several surrounding rural counties (RUCCs = 4–7) (21) south of the clinic, and rural census tracts, using Federal Office of Rural Health Policy definitions for rural zip codes in urban counties (22), situated north and west of the clinic.

Data collection occurred over 3 weeks in December 2019 (23), with 267 patients enrolled in the parent study. In the weeks before data collection, OTP staff informed patients of the study and distributed recruitment materials. Research staff were onsite for data collection three varied days of each week. A convenience sample of patients completed self-administered computer-based surveys in a private room located at the clinic. Research staff obtained informed consent (using an information sheet approved with a documentation waiver), assisted with surveys as needed (e.g., due to difficulties with reading or technology), and provided compensation ($25 gift card to a large shopping outlet) for completing surveys.

For this analysis, we focus on the subset of patients who had their treatment funded by Medicaid or Medicare. All patients were eligible for this analysis, regardless of when they started treatment. Thus, the sample includes patients new to treatment, as well as those who were engaged with the clinic for long-term care. We focus on the analytic sample of Medicaid/Medicare beneficiaries receiving methadone treatment^1^ because preliminary analyses demonstrated higher rates of comorbid disorders among Medicaid/Medicare beneficiaries compared to patients reporting private health insurance or self-pay. This is consistent with literature showing a greater comorbidity burden among publicly-insured populations with OUD (24). Additionally, we were not adequately powered to compare differences based on payment type in the analytic sample (*N* = 210; Medicaid: *n* = 196, 93.3%; Medicare: *n* = 14, 6.7%). All study procedures were approved by the Wayne State University Institutional Review Board.

### Measures

Surveys assessed measures across three domains: demographic characteristics, opioid use characteristics, and comorbid affective and substance use disorder screening measures.

#### Demographic Characteristics

Patients provided demographic information, including their current age (years), geography (zip code of residence), gender identity (male, female, other), race/ethnicity (Arabic/Middle Eastern, Hispanic/Latino, Non-Hispanic African American, Non-Hispanic Asian, Non-Hispanic Native American, Non-Hispanic Native Hawaiian, Non-Hispanic White, Non-Hispanic more than one race), educational attainment (earned high school diploma or GED, had not earned a high school diploma or GED), and their public health insurance type (Medicaid, Medicare). To measure rural-urban community of residence, we used the Goldsmith Modification (25), a technique outlined by the Federal Office of Rural Health Policy to categorize rurality using patient zip codes at the county (RUCCs 4–9) and federally-defined rural census tracts (i.e., rural zip codes embedded within urban counties). Though the gender item included a non-binary response option, no patients reported a gender aside from male or female. Because the sample was predominantly Non-Hispanic White and other race/ethnicity groups were smaller (<5%), race/ethnicity was dichotomized (Non-Hispanic White, other race/ethnicity) to ensure adequate statistical power to detect group differences.

#### Opioid Use Characteristics

Patients answered questions about five opioid use characteristics. These brief measures were selected due to high clinical feasibility and reduced patient burden. First, patients were asked about non-medical opioid use in the past year. Past-year non-medical opioid use was then compared with the date the patient started their current treatment episode to indicate the absence of past-year use (reference category) or occurrence of past-year use before starting treatment or while in treatment (coded as 1 and 2). Patients reported their history of fentanyl use, whether intentional or unintentional (dichotomous: no history, history), as well as their preference for injection drug use (dichotomous: not preferred, preferred). Patients also completed an adapted version of the Heroin Use Consequence scale (26) to assess lifetime opioid use consequences. Though the original HUC scale focuses on consequences of heroin use specifically, in the current study patients were asked to consider their use of all opioids except those used as directed by a doctor. Education-related consequences (three items from the original 20-item scale), that were among the least endorsed in the scale development study (26), were excluded to reduce time burden. This resulted in a total of 17 items that were summed. Last, we assessed whether patients had been in treatment during the current episode for 1 year or more.

#### Comorbid Affective and Substance Use Disorder Screening Measures

Validated screens were administered for seven comorbid disorders. Established cut scores were used to categorize patients as having probable comorbid disorders (i.e., positive screens). Three screens assessed probable comorbid affective disorders, including depression (Patient Health Questionaire-2; PHQ-2, a score of three or greater during the past 2 weeks was interpreted as a positive screen) (27), anxiety (Generalized Anxiety Disorder 2-Item Scale; GAD-2, a score of three or greater during the past 2 weeks was interpreted as a positive screen) (27), and PTSD (Primary Care PTSD Screen for DSM-5; PC-PTSD-5, a score of four or greater during the past month was interpreted as a positive screen) (28). Four screens assessed probable comorbid substance use disorders during the past year, including stimulants (Stimulant Severity of Dependence Scale, a score of three or greater was used) (29), cannabis (Cannabis Severity of Dependence scale, a score of three or greater was used) (30), alcohol (AUDIT-C, for women, a score of three or greater was used and for men a score of four or greater was used to identify patients with hazardous drinking or active alcohol use disorder) (31), and sedatives (Sedative Severity of Dependence Scale, a score of six or greater was used) (29).

## Data Analysis

We analyzed data using SPSS version 27 (IBM Corp., 2017). After the removal of patients from the analytic sample (described in Settings and Procedure), cases from variables with small amounts of missing data (1–2 cases) were removed using listwise deletion by default. Little's MCAR test indicated data were missing completely at random [χ2 (158) = 169.586, *p* = 0.250] (32). Measures of central tendency and distribution were calculated for all variables. Group (*t*-tests, chi-square, one-way ANOVA) and correlation analyses (Kendall's Tau-b) explored differences and associations between demographic characteristics, opioid use characteristics, and comorbid disorder screening measures. Adjusted standardized residuals (ASRs) were used to estimate low (≤-2) and high values (≥2), in line with Haberman's rule of thumb (33), in bivariate analyses of past-year non-medical opioid use status (a three-level categorical variable). We then conducted binomial logistic regressions to examine whether past-year non-medical opioid use status remained a significant differentiator of probable comorbid disorders after including demographic and opioid use characteristics that demonstrated directional (*p* < 0.20) associations in bivariate analyses. Since the comorbid disorder screening measures for alcohol and depression did not differ by past-year non-medical opioid use status in bivariate analyses, they were not included alongside the other five comorbid disorder screening measures as dependent variables in multivariable regression analyses. Due to collinearity with past-year non-medical opioid misuse status, time in treatment was excluded from multivariable analyses.

## Results

### Sample Description

Table 1 displays descriptive information about the analytic sample for demographic characteristics, opioid use characteristics, and rates of comorbid disorders.

**Table 1:** Sample characteristics (*N* = 210).

	***n***	**Valid %**	***M* (SD)**
**Demographic characteristics**
Age (in years)			38.53 (10.13)
Female gender	130	62.2	
High school degree or equivalent	158	75.2	
Non-Hispanic White race/ethnicity	180	86.1	
Rural community	29	14.4	
**Opioid use characteristics**
Fentanyl use	129	61.7	
Injection preference	83	39.5	
Opioid use consequences			10.25 (4.49)
Time in treatment > 1 year	169	71.6	
**Comorbid disorders**
Depression	85	41.1	
Anxiety	100	48.1	
PTSD	70	33.7	
Alcohol	31	14.9	
Marijuana	40	19.0	
Stimulant	58	27.6	
Sedative	16	7.6	

*Age range, 22–72 years. Frequencies for other race/ethnicity groups: Non-Hispanic more than one race (n = 10, 4.8%), Non-Hispanic African American or Black (n = 8, 3.8%), Hispanic any race (n = 7, 3.3%), Non-Hispanic Asian American (n = 2, 1/0%), and Non-Hispanic Native American or Alaska Native (n = 2, 1.0%). Reference groups (in parentheses) were as follows: gender (male), education (less than HS/GED), race (other race/ethnicity), community (non-rural), fentanyl use (no history), and injection opioid use (no history)*.

### Group Differences for Comorbid Disorders by Demographic and Opioid Use Characteristics

Table 2 presents bivariate relationships between demographic and opioid use characteristics and each of the seven comorbid disorder screening measures. Younger age was associated with PTSD (*p* < 0.001), and lower educational attainment was associated with sedative use disorder (*p* < 0.05). Other demographic characteristics met bivariate determination (*p* < 0.20) for multivariable regression analyses, including the negative relationships between female gender and marijuana use disorder and the positive relationship between female gender and anxiety and alcohol use disorder, and the positive relationship between living in a rural community and marijuana use disorder. The Non-Hispanic White race/ethnicity variable was excluded from the sedative use disorder model in bivariate and multivariable analyses due to perfect separation (i.e., 100% of the patients with sedative use disorder reported non-Hispanic White race/ethnicity).

**Table 2:** Group differences for comorbid disorders by demographic and opioid use characteristics.

	**Depression**	**Anxiety**	**PTSD**	**Alcohol**	**Marijuana**	**Stimulant**	**Sedative**
**Demographic characteristics**							
Age	*τ_*b*_* = −0.004	*τ_*b*_* = −0.050	*τ_*b*_* = −0.217	*τ_*b*_* = 0.016	*τ_*b*_* = −0.014	*τ_*b*_* = 0.024	*τ_*b*_* = −0.043
	*p* = 0.951	*p* = 0.386	*p* = 0.000	*p* = 0.791	*p* = 0.808	*p* = 0.682	*p* = 0.455
Female gender	*t* = 0.234	*t* = 1.479	*t* = 0.818	*t* = 1.423	*t* = −1.561	*t* = −0.145	*t* = 0.002
	*p* = 0.815	*p* = 0.141	*p* = 0.414	*p* = 0.156	*p* = 0.120	*p* = 0.885	*p* = 0.980
High school degree or equivalent	*t* = −0.672	*t* = −1.121	*t* = 0.735	*t* = −0.703	*t* = −1.259	*t* = −0.227	*t* = −0.168
	*p* = 0.502	*p* = 0.264	*p* = 0.463	*p* = 0.483	*p* = 0.209	*p* = 0.821	*p* = 0.015
Non-Hispanic White race/ethnicity	*t* = −0.014	*t* = 1.025	*t* = −0.227	*t* = −0.927	*t* = −0.228	*t* = 0.407	*excluded from the model^*a*^*
	*p* = 0.989	*p* = 0.306	*p* = 0.820	*p* = 0.355	*p* = 0.820	*p* = 0.685	
Rural community	*t* = −0.268	*t* = −0.711	*t* = 0.481	*t* = −0.184	*t* = 1.707	*t* = −0.402	*t* = 0.046
	*p* = 0.789	*p* = 0.478	*p* = 0.631	*p* = 0.854	*p* = 0.191	*p* = 0.688	*p* = 0.519
**Opioid use characteristics**							
Fentanyl use	*t* = 0.234	*t* = 1.794	*t* = 2.769	*t* = 0.391	*t* = 1.067	*t* = 2.193	*t* = 0.153
	*p* = 0.815	*p* = 0.074	*p* = 0.006	*p* = 0.697	*p* = 0.287	*p* = 0.029	*p* = 0.028
Injection preference	*t* = 1.127	*t* = 0.592	*t* = −0.178	*t* = 1.167	*t* = −0.290	*t* = 0.338	*t* = 0.098
	*p* = 0.261	*p* = 0.555	*p* = 0.859	*p* = 0.244	*p* = 0.772	*p* = 0.736	*p* = 0.155
Opioid use consequences	*τ_*b*_* = 0.079	*τ_*b*_* = 0.173	*τ_*b*_* = 0.254	*τ_*b*_* = −0.028	*τ_*b*_* = 0.094	*τ_*b*_* = 0.229	*τ_*b*_* = 0.170
	*p* = 0.178	*p* = 0.002	*p* = 0.000	*p* = 0.605	*p* = 0.121	*p* = 0.000	*p* = 0.004

*Bivariate analyses use Kendall's tau-b (τ_b_) and t-tests. Listwise n = 200. Alpha threshold (p < 0.20, two-tailed) used for bivariate determination procedures in multivariable models. Reference groups (in parentheses) were as follows: gender (male), education (less than HS/GED), race (other race/ethnicity), community (non-rural), fentanyl use (no history), and injection opioid use (no history)*.

a *Race/ethnicity variable was excluded from the sedative use disorder model analysis due to perfect separation (100% of patients with sedative use disorder reported Non-Hispanic White race/ethnicity)*.

With regard to opioid use characteristics, a greater lifetime accumulation of opioid use consequences was positively associated with PTSD (*p* < 0.001), stimulant use disorder (*p* < 0.001), anxiety (*p* < 0.01), sedative use disorder (*p* < 0.01), and met bivariate determination (*p* < 0.20) for marijuana use disorder and depression. A history of fentanyl use was associated with PTSD (*p* < 0.01), sedative use disorder (*p* < 0.05), and stimulant use disorder (*p* < 0.05). A preference for injection drug use was unrelated to all comorbid disorder screens, but did meet bivariate determination (*p* < 0.20) for sedative use disorder.

### Group Differences for Comorbid Disorders by Past-Year Non-Medical Opioid Use Status

Table 3 displays bivariate analyses for comorbid disorder screening measures by past-year non-medical opioid use status. Five of the seven comorbid disorders, including anxiety (*p* < 0.05), PTSD (*p* < 0.001), marijuana use disorder (*p* < 0.05), stimulant use disorder (*p* < 0.001), and sedative use disorder (*p* < 0.01) differed significantly by past-year non-medical opioid use status, whereas depression and alcohol use disorder were unrelated. High and/or low ASR values were examined for the same five comorbid disorders among the three past-year non-medical opioid use status groups. Specifically, patients reporting no past-year non-medical opioid use had low levels (ASR ≤-2) of anxiety, PTSD, marijuana use disorder, stimulant use disorder, and sedative use disorder. In contrast, patients reporting past-year non-medical opioid use that occurred before treatment had high levels (ASR ≥2) of PTSD, marijuana use disorder, and stimulant use disorder, while patients reporting past-year non-medical opioid use while in treatment had high levels (ASR ≥2) of stimulant use disorder and sedative disorder.

**Table 3:** Group differences for comorbid disorders by past-year non-medical opioid use status.

	**Depression**	**Anxiety**	**PTSD**	**Alcohol**	**Marijuana**	**Stimulant**	**Sedative**
No PY non-medical opioid use (*n* = 113)	37.3 (41)	38.9 (43)^a^	23.2 (26)^a^	10.8 (12)	12.4 (14)^a^	12.4 (14)^a^	1.8 (2)^a^
PY non-medical opioid use, before tx (*n* = 29)	44.8 (13)	62.1 (18)	62.1 (18)^b^	20.7 (6)	34.5 (10)^b^	44.8 (13)^b^	13.8 (4)
PY non-medical opioid use, while in tx (*n* = 68)	45.6 (31)	57.4 (39)	38.8 (26)	19.1 (13)	23.5 (16)	45.6 (31) ^b^	14.7 (10)^b^
Total sample	41.1 (85)	48.1 (100)	33.7 (70)	14.9 (31)	19.0 (40)	27.6 (58)	7.6 (16)
	*χ^2^* = 1.398, *p* = 0.497	*χ^2^* = 8.496, *p* = 0.014	*χ^2^* = 16.75 *p* < 0.001	*χ^2^* = 3.184, *p* = 0.204	*χ^2^* = 8.615, *p* = 0.013	*χ^2^* = 28.390, *p* < 0.001	*χ^2^* = 11.915, *p* = 0.003

*PY, past-year; tx, treatment. All values reported are “valid % (n)” unless otherwise noted*.

a *Adjusted Standardized Residual (ASR) = ≤-2*.

b *Adjusted Standardized Residual (ASR) = ≥2*.

### Multivariable Regressions of Comorbid Disorders

Five separate multivariable regression models were conducted (see Table 4). Four models were significant (PTSD: *p* < 0.001; stimulant use disorder: *p* < 0.001; sedative use disorder: *p* < 0.001; anxiety: *p* < 0.01), and one was not (marijuana use disorder: *p* > 0.05).

**Table 4:** Multivariable regressions of comorbid disorders.

**Variable**	**Anxiety**	**PTSD**	**Marijuana**	**Stimulant**	**Sedative**
PY non-medical opioid use, before tx	*B* = 0.737, *p* = 0.113	*B* = 1.478, *p* = 0.003^**^	*B* = 1.024, *p* = 0.056	*B* = 1.373, *p* = 0.006^**^	*B* = 1.912, *p* = 0.042^*^
	95% CI = 0.84^−^5.20	95% CI = 1.67^−^11.52	95% CI = 0.97^−^7.96	95% CI = 1.50^−^10.42	95% CI = 1.07^−^42.75
PY non-medical opioid use, while in tx	*B* = 0.603, *p* = 0.072	*B* = 0.372, *p* = 0.314	*B* = 0.588, *p* = 0.169	*B* = 1.572, *p* < .001^***^	*B* = 2.116, *p* = 0.012^*^
	95% CI = 0.95^−^3.53	95% CI = 0.70^−^2.99	95% CI = 0.78^−^4.17	95% CI = 2.24^−^10.38	95% CI = 1.61^−^42.88
Age	Not in model	*B* = −0.059, *p* = 0.003^**^	Not in model	Not in model	Not in model
		95% CI = 0.91^−^0.98			
Female gender identity	*B* = 0.644, *p* = 0.038^*^	Not in model	*B* = −0.455, *p* = 0.634	Not in model	Not in model
	95% CI = 1.04^−^3.50		95% CI = 0.30^−^1.33		
High school degree or equivalent	Not in model	Not in model	Not in model	Not in model	*B* = −1.458, *p* = 0.012^*^
					95% CI = 0.07^−^0.73
Non-Hispanic White race/ethnicity	Not in model	Not in model	Not in model	Not in model	Excluded from analysis^*a*^
Rural community	Not in model	Not in model	*B* = 0.608, *p* = 0.478	Not in model	Not in model
			95% CI = 0.72^−^4.69		
Fentanyl use	*B* = 0.209, *p* = 0.541	*B* = 0.121, *p* = 0.753	Not in model	*B* = −0.019, *p* = 0.963	*B* = 0.544, *p* = 0.525
	95% CI = 0.63^−^2.41	95% CI = 0.53^−^2.40		95% CI = 0.45^−^2.16	95% CI = 0.32^−^9.25
Injection preference	Not in model	Not in model	Not in model	Not in model	*B* = −0.147, *p* = 0.801
					95% CI = 0.37^−^3.64
Opioid use consequences	*B* = 0.067, *p* = 0.074	*B* = 0.112, *p* = 0.009^**^	*B* = 0.020, *p* = 0.663	*B* = 0.110, *p* = 0.019^*^	*B* = 0.146, *p* = 0.111
	95% CI = 0.99^−^1.15	95% CI = 1.03^−^1.22	95% CI = 0.93^−^1.12	95% CI = 1.02^−^0.23	95% CI = 0.97^−^1.39
Model metrics	**χ**^2^(5) = 16.931	**χ**^2^(5) = 39.991	**χ**^2^(5) = 8.736	**χ**^2^(4) = 34.324	**χ**^2^(6) = 25.126
	*R*^2^ = 0.105, *p* = 0.005	*R*^2^ = 0.243, *p* < .001	*R*^2^ = 0.069, *p* = 0.120	*R*^2^ = 0.222, *p* < .001	*R*^2^ = 0.271, *p* < .001

* *p < 0.05*.

** *p < 0.01*.

*** *p < 0.001*.

*PY, past-year; tx, treatment. Variables not in model did meet alpha threshold (p < 0.20, two-tailed) in bivariate analyses of comorbid psychiatric disorders. No PY non-medical opioid use was the reference group for PY non-medical opioid use, before tx and PY non-medical opioid use, while in tx*.

a *Race/ethnicity variable was excluded from the sedative use disorder model analysis due to perfect separation (100% of patients with sedative use disorder reported Non-Hispanic White race/ethnicity). All R^2^ were Nagelkerke R Square values*.

Past-year non-medical opioid use before treatment was significant in three models (PTSD: *p* < 0.01; stimulant: *p* < 0.01; sedative: *p* < 0.05). Past-year non-medical opioid use while in treatment was significant in two models (stimulant: *p* < 0.001; sedative: *p* < 0.05). A greater number of opioid use consequences was significant in two models (PTSD: *p* < 0.01; stimulant: *p* < 0.05). Other significant variables included younger age in the PTSD model (*p* < 0.01), lower educational attainment in the sedative model (*p* < .05), and female gender in the anxiety model (*p* < 0.05). Fentanyl use, injection drug use preference, Non-Hispanic White race/ethnicity, and rural community were not significant in any models.

## Discussion

This study identified rates and correlates of comorbid disorders among Medicaid/Medicare beneficiary patients from small urban and rural communities receiving methadone treatment for OUD. This analysis highlights a high comorbidity burden, especially for affective disorders. Our findings also reveal a consistent role for past-year non-medical opioid use to detect patients in greater need of integrated care for comorbid disorders. To date, few studies have examined comorbid affective and substance use disorders among this understudied population.

Comorbid disorders were common in this sample, especially for affective disorders, with rates of 48, 41, and 34%, for anxiety, depression, and PTSD, respectively. Consistent with predictions, rates were lower for comorbid substance use disorders, though 28% of patients still screened positive for stimulant use disorder. When analyzing differences by past-year non-medical opioid use status, considerably higher rates for all seven comorbid disorders were demonstrated among patients reporting use before and/or while in treatment. As illustration, within the sub-sample of patients reporting past-year use that occurred before treatment, or while in treatment, 62 and 57%, respectively, screened positive for anxiety, compared to 39% of patients reporting no past-year use. Similarly, 62% of patients reporting past-year use while in treatment screened positive for PTSD, compared to 39% reporting past-year before treatment, and 23% reporting no past-year use. The difference was starkest for stimulant use disorder, where 46% (past-year use while in treatment) and 45% (past-year use before treatment), respectively, screened positive, compared to only 12% of patients reporting no past-year use. For all seven disorders assessed, patients reporting no past-year use had lower rates than either group reporting past-year use, regardless of whether it occurred before or while in treatment. Consistent with hypotheses, these relationships remained even when accounting for other demographic and opioid use characteristics, as past-year non-medical opioid use was the variable most consistently associated with comorbid disorders in multivariable analyses. This relationship was strongest for PTSD, stimulant use disorder, and sedative use disorder.

One other opioid use characteristic, opioid use consequences, was associated with an increased likelihood of having comorbid disorders. In bivariate analyses, a greater number of consequences was associated with higher rates for anxiety, PTSD, stimulant use disorder, and sedative use disorder, though in multivariable analyses, significant associations only remained for PTSD and stimulant use disorder. This finding extends prior work demonstrating that a greater accumulation of opioid use consequences increases the likelihood of comorbid affective disorder symptoms among people who regularly use heroin in a large urban area in the same state as this study (34). Injection use preference was not related to comorbid disorder status, despite prior research demonstrating an association between injection use and comorbid affective and substance use disorders among participants in large urban areas (35, 36). Similarly, fentanyl use was not related to comorbid disorders. Our analysis may highlight that even though fentanyl penetration to the drug supply and comorbidity are key contributors to overdose risk among Medicaid beneficiaries (37), in this geographic setting, the relationship of fentanyl use and comorbidity may be better understood as an interactive (vs. probabilistic) relationship. With regard to demographic characteristics, few relationships were observed. Younger age, lower educational attainment, and female gender were associated with a greater likelihood of PTSD, stimulant use disorder, and anxiety, respectively. Comorbid disorder status was unrelated to race/ethnicity in this sample. Similarly, rates did not differ for patients from rural areas compared to those residing in the small urban area where the OTP is located, suggesting that comorbid disorders among rural and small urban patients may be more similar than different.

The findings from this study highlight a few clinical implications. First, we suggest treatment authorities require OTPs to use validated screening tools for comorbid disorders, going a step further than current federal guidelines (9, 10). Second, we recommend that capacity and planning for coordinated care, particularly for affective disorders, be built into existing intake procedures. These strategies might include providing co-located services for affective disorders (15), developing partnerships for mental health service provision that build in accommodations for people living in small urban and rural areas (38), or evaluating the efficacy of evidence-based approaches for affective disorders, such as the Unified Protocol or Acceptance and Commitment Therapy (39, 40), when implemented through telehealth or adapted as computer-delivered interventions. Regardless of the strategy to assess and coordinate care, approaches should emphasize feasible innovations that mitigate implementation barriers and present financially sustainable changes at OTPs to facilitate adoption in a complex treatment system. The use of mixed methods implementation science protocols, such as NIATx (41), adapted to OTP settings in small urban and rural settings, may be a promising strategy to identify a more comprehensive understanding of patient and provider experiences as a means of improving and sustaining innovations to existing care models. This may be especially important at treatment intake, given that nearly all patients (e.g., transfers excluded), initiate methadone treatment following recent and ongoing non-medical opioid use, a robust correlate of affective disorder comorbidity in this sample. Third, we recommend future research that examines whether routinely screening patients (e.g., during clinical sessions and/or through short message services) engaged in long-term treatment for recent non-medical opioid use is feasible and improves linkage to care for comorbid disorders. Findings may provide support for the one-item measure presented here as a more efficient and less invasive method than current protocols (e.g., urine drug screens). Last, polysubstance use of stimulants and opioids has been rising nationally, and is a key determinant in the fourth wave of the opioid overdose crisis (42). While this study didn't directly assess polysubstance use, the high rates of comorbid stimulant use disorder in this sample of OUD patients suggests polysubstance use is occurring for a sizable portion of patients, who urgently need integrated approaches that can reduce overdose-related harm.

This study has limitations. First, the sample was a convenience sample with a heterogeneous length of care for their current treatment episode. While this does introduce important differences, many studies examine recent non-medical opioid use without inquiring about treatment engagement. Furthermore, we sought to counteract this heterogeneity and aid clinical interpretation by providing comorbid disorder rates within sub-samples categorized by past-year non-medical opioid use status. Second, we did not assess all possible comorbid affective and substance use disorders, in part due to time constraints to gather info on disorders with low base rates (e.g., schizophrenia, hallucinogen use disorder). Similarly, we did not assess addictions not commonly addressed at OTPs (e.g., tobacco use disorder, gambling disorder), nor did we assess comorbid health conditions, such as infectious diseases, that overlap with OUD (43), and represent other important avenues where integrated approaches improve treatment outcomes (44). Future studies should investigate a full spectrum of OUD-related comorbidities, which ostensibly would highlight an even higher comorbidity burden and need for integrated approaches than this analysis. Third, our sample, while innovative in many ways (small urban and rural setting, public insurance homogeneity), was not powered to conduct an in-depth comparison of racial differences or population-specific comorbidity patterns (e.g., comorbid disorder rates among Black/African American patients), which may have provided valuable information about health disparities. Future research should gather data in small urban and/or rural settings where there is a greater representation of Medicaid/Medicare beneficiaries from diverse racial groups (e.g., Black/African Americans in the Deep South, Hispanic/Latinos in the American Southwest, and Native Americans in the Great Plains) who are receiving methadone treatment. Last, our comorbid affective disorder screening measures, while using established administration instructions and timelines, did not assess whether the patient would've screened positive at other time points in the past-year. As a result, our analysis may underestimate the rates of affective disorders compared to other studies (3, 45).

In conclusion, this study highlights a high rate of comorbid disorders, especially affective disorders, among publicly-insured methadone patients from small urban and rural areas. This burden is especially high for patients reporting recent non-medical opioid use, regardless of whether that use occurred before or during their current methadone treatment episode. Innovative and feasible approaches that assess patients for comorbid disorders and recent non-medical opioid use are needed to improve care coordination. We encourage local and federal treatment authorities, OTP directors, and methadone treatment researchers to consider our findings when developing screening, implementation, and coordinated care strategies.

## Data Availability Statement

The dataset presented in this article are not readily available because participants of this study did not agree for their data to be shared publicly, so supporting data is not available. Requests to access the dataset should be directed to JL (jlister@ssw.rutgers.edu), SR (stella@wayne.edu), and EA (ad2634@wayne.edu).

## Ethics Statement

This study involved human participants and was reviewed and approved by the Wayne State University Institutional Review Board. Participants read an information sheet prior to participating in this study. Written informed consent was not required, as a waiver of documentation was obtained.

## Author Contributions

JL, GL, JE, EP, EA, and SR: led writing, conceptualization, and methodology. JL and JE: oversaw data analyses. GL: led data analyses. EA and SR: provided reviews. All authors contributed to the article and approved the submitted version.

## Funding

Data collection was supported by funds from the Substance Abuse and Mental Health Services Administration to the Michigan Department of Health and Human Services under Grant TI080228 (to EA). Funds from a Wayne State University School of Social Work Research Enhancement Program (to JL) supported pilot data, feasibility analysis, and clinic partnerships that informed study design.

## Conflict of Interest

The authors declare that the research was conducted in the absence of any commercial or financial relationships that could be construed as a potential conflict of interest.

## Publisher's Note

All claims expressed in this article are solely those of the authors and do not necessarily represent those of their affiliated organizations, or those of the publisher, the editors and the reviewers. Any product that may be evaluated in this article, or claim that may be made by its manufacturer, is not guaranteed or endorsed by the publisher.

## Footnotes

^1^Forty cases were excluded when treatment was funded by private health insurance (*n* = 34) or self-pay (*n* = 6). An additional 17 cases were removed for missing data on key study variables (past-year non-medical opioid use, current methadone status).
